# Quantitative EEG features selection in the classification of attention and response control in the children and adolescents with attention deficit hyperactivity disorder

**DOI:** 10.4155/fsoa-2017-0138

**Published:** 2018-02-14

**Authors:** Azadeh Bashiri, Leila Shahmoradi, Hamid Beigy, Behrouz A Savareh, Masood Nosratabadi, Sharareh R N Kalhori, Marjan Ghazisaeedi

**Affiliations:** 1 Department of Health Information Management, School of Allied Medical Sciences, Tehran University of Medical Sciences, Tehran, Iran; 2 Department of Computer Engineering, Sharif University of Technology, Tehran, Iran; 3 Student Research Committee, School of Allied Medical Sciences, Shahid Beheshti University of Medical Sciences, Tehran, Iran; 4 Department of Clinical Psychology, University of Social Welfare & Rehabilitation Sciences, Tehran, Iran

**Keywords:** ADHD, IVA-CPT, QEEG

## Abstract

**Aim:**

Quantitative EEG gives valuable information in the clinical evaluation of psychological disorders. The purpose of the present study is to identify the most prominent features of quantitative electroencephalography (QEEG) that affect attention and response control parameters in children with attention deficit hyperactivity disorder.

**Methods:**

The QEEG features and the Integrated Visual and Auditory-Continuous Performance Test ( IVA-CPT) of 95 attention deficit hyperactivity disorder subjects were preprocessed by Independent Evaluation Criterion for Binary Classification. Then, the importance of selected features in the classification of desired outputs was evaluated using the artificial neural network.

**Results:**

Findings uncovered the highest rank of QEEG features in each IVA-CPT parameters related to attention and response control.

**Conclusion:**

Using the designed model could help therapists to determine the existence or absence of defects in attention and response control relying on QEEG.

## Lay abstract

A model has been developed to identify the most prominent features of QEEG, and electrophysiological monitoring method, that affect attention and response control parameters in the IVA-CPT test, a software that helps clinicians evaluate attention deficit hyperactivity disorder. The designed model can help therapists to determine the existence or absence of defects in attention and response control.

Attention deficit hyperactivity disorder (ADHD) is one of the most common neurobehavioral disorders, diagnosed in 3–7% of school-age children (age 5–9 years) [1 3]. ADHD as a developmental disorder is accompanied by depression, conduct disorder, anxiety and learning disabilities [4,5]. This disorder is usually diagnosed when the nature, frequency and duration of the behavioral symptoms such as inattention results changes in the individual's functions. These changes are according to the defined criteria of the Diagnostic and Statistical Manual of Mental Disorders (DSM-IV) and the International Classification of Mental and Behavioral Disorders – 10th revision [6 8]. The most important symptom in children with ADHD is inability to pay attention, meaning they often make mistakes in listening to others, following instructions, finishing tasks or maintaining personal belongings. To control and improve the ADHD rehabilitation, the measurement of attention in the children with ADHD, especially during primary school, can be helpful [9–11].

Recently, IVA-CPT as a computerized test has been designed according to DSM-IV criteria to evaluate two universal scales (Scale Response Control and Scale Attention Quotient) in individuals with ADHD [6,11–13]. IVA-CPT measures different kinds of attention including alternating attention, sustained attention, divided attention, selective attention, focused attention and also full attention and full response control, separately. Also, this test measures other related parameters of attention and response control, including vigilance, focus, speed, prudence, consistency and stamina in both visual and auditory aspects. IVA-CPT as a diagnostic supplementary tool can help therapists to make an accurate diagnosis and differentiate between the subtypes of ADHD in individuals aged 6 years and up [6,9–11,14–17].

Despite the benefits of IVA-CPT, it is a time-consuming test and may only identify individuals with high risk of attention deficit. On the other hand, the different levels in the individuals’ computer skills, their motivation and mood as well as their ability to understand the structure of the test may unpredictably affect the test results. In addition, this test is a quick evaluation of a short period of time and cannot necessarily represent a general clinical experience of the individuals with ADHD. Also, it sometimes has a placebo effect that temporarily affects the level of arousal of individuals and leads to less accurate results [18,19].

Studies have shown that IVA-CPT can be integrated with the EEG, which is one of the diagnostic indicators for ADHD and can evaluate clinical status of individuals with ADHD [9,20–22]. EEG signals are produced as a result of neural activity and, according to their frequency, are divided into categories Delta (0.5–4 Hz), Theta (4–8 Hz), Alpha (8–13 Hz), Beta (13- 30 Hz) and Gamma (>30 Hz) frequency bands [23]. Each frequency band is responsible for a special behavior [24] and its fluctuations are largely related to various mental conditions and psychological disorders such as learning disabilities, damaged brain and ADHD [14]. Modern computer systems, by applying spectral analysis, are able to quantitatively map the EEG signals [25]. With EEG, abnormal patterns of brain activity are visible easily. But, other patterns that are not visible with the eye can be seen through the computer. QEEG, the use of a computer to extract information from brain waves, is compared with traditional approaches for tracking EEG on paper. Based on findings from previous studies, QEEG gives us valuable information about the various aspects of the functions of the brain waves and so is suitable for clinical evaluation of neuropsychiatric conditions, such as ADHD [14,26,27]. The purpose of the present study is therefore to identify the most prominent features of QEEG in the classification of attention and response control parameters by correlation the QEEG with the IVA-CPT in the children and adolescents with ADHD.

## Materials & methods

**Figure 1.**
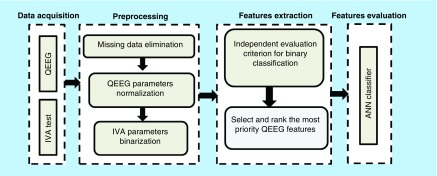
**Flowchart of methodology.** ANN: Artificial neural network; IVA: Integrated visual and auditory; QEEG: Quantitative electroencephalography.

The methodology of this study is summarized in Figure 1. It consists of four main phases, including data acquisition from QEEG and IVA-CPT, preprocessing, feature selection and features evaluation.

### Participants

In the present study, 95 ADHD subjects with ages 7 through 18 years old participated. Participants visited in two rehabilitation clinics in Tehran and Mashhad from 2013 to 2016. Based on the clinical evaluation by psychologists and psychiatrists, they had met the diagnostic criteria of the DSM-IV [28], to include in the ADHD diagnosis. Table 1 shows the demographic characteristics of participants based on gender, the range of ages and the type of ADHD. The subjects who had a history of other problematic conditions such as prenatal disorders, head injury, convulsive disorders, CNS diseases and other neurological disorders, were excluded from the study.

**Table 1.** **The demographic characteristics of subjects.**

**Participants**		**Children and adolescents with ADHD**									

		**Hyperactive impulsive**		**Inattentive**		**Combination**		**Unspecified**		**Total**	

		**Number**	**Percent**	**Number**	**Percent**	**Number**	**Percent**	**Number**	**Percent**	**Number**	**Percent**
Sex	Female	5	33%	7	23%	11	25%	0	0%	23	24%

	Male	10	67%	23	77%	33	75%	6	100%	72	76%

Age	7–12	9	60%	20	67%	22	50%	4	67%	55	58%

	13–18	6	40%	10	33%	22	50%	2	33%	40	42%

ADHD: Attention deficit hyperactivity disorder.

### Data acquisition

#### QEEG

In this phase, before doing EEG test, ADHD subjects took the following steps: washing hair the night before the test and not using any products like sprays or gels; stop taking any medications before the test according to the physician opinion (such as stop taking Ritalin 24 h prior [all ADHD samples in this study, did not take any stimulant drugs before doing the EEG test and thus there was no effect of used drugs on the results]), and also informing the technician performing the EEG of these medications; avoid eating or drinking caffeine at least 8 h before the EEG test; having enough sleep night before the test and other recommendations that depend on the situation of taking EEG, for example, in the rest or task condition. The EEG of participants was measured by using MITSAR with 19-electrode channels. The reference electrode was linked to ears (A1+A2/2). After removing noise of all channels, the sampling rate for acquiring data was adjusted in 250 Hz. The EEG was taken in a situation in which the subjects were relaxed and with closed eyes. The elastic cap with 19 tin electrodes located according to the international 10–20 system in Fp1, Fp2, F3, F4, F7, F8, Fz, C3, C4, Cz, T3, T4, T5, T6, P3, P4, Pz, O1 and O2 sites [29]. Also Ten20 as an opaque adhesive paste was applied to attach cup electrodes and reduce skin impedance. The conversion of EEG to quantitative EEG was done by NeuroGuide software. Finally, the QEEG of 95 ADHD subjects were acquired. The QEEG file of each participant included an excel file of QEEG features and a set of bmp file of brain maps. In order to centralize excel files of participants, a program was developed in MATLAB software. Data aggregation with MATLAB software resulted in a dataset in which the rows were ADHD samples and the columns represent QEEG features as independent variables (95*9961).

#### IVA-CPT

After the QEEG, the IVA-CPT was administered to ADHD subjects. IVA-CPT provides quotient scores into four groups consist of response control, attention, attribute and symptomatic [17]. In our study, IVA-CPT data about response control and attention have been considered. The full scale response control in separate visual and auditory dimensions is divided to prudence, consistency and stamina scales. Also, the full scale attention in separate visual and auditory dimensions is divided to vigilance, focus and speed scales. Also, the scales of different type of attention were considered. In general, 28 IVA-CPT parameters were extracted manually from the IVA-CPT reports (Table 3) and stored for each participant in addition to QEEG data. For entire process, the MATLAB software (2016 b version) was used to write a program consisting of a number of functions. Each function had a special task and was part of the overall process.

### Preprocessing

#### Removing missing data

Missing data means there are no data values for the variable [30,31]. The solutions to handle missing data included predicting, fitting or deleting missing values. Due to the complexity of the relationship between QEEG features, prediction or fitting was not possible [32], therefore, in spite of the loss of data, our approach inevitably was to remove records containing missing values.

#### QEEG parameters normalization

One of the preprocessing stages is normalization. Normalization is a scaling for the prediction or forecasting purpose. Because many of the machine learning techniques have better performance with normal data, input data (QEEG section) were transformed into the 0 to 1 (0.1) range according to the following equation:
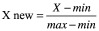



Equation 1. Normalization

#### IVA-CPT parameters binarization

Because of the insufficient samples for fitting, the binarization was performed to find the absence or present of defect in IVA-CPT parameters. They were binarized according to the interpretation manual of IVA-CPT [17]. According to interpretation manual of IVA-CPT, obtained quotient score below 90 in each parameter means the existence of defect in it. So, we organized output as follows:
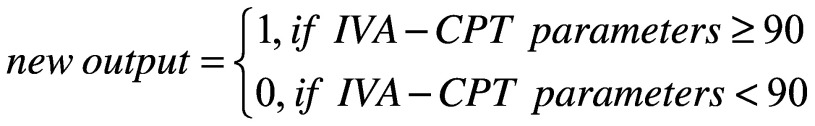



Equation 2. IVA-CPT parameters binarization

To overcome class imbalance, the adaptive synthetic sampling approach was applied. The adaptive synthetic sampling approach algorithm is built based on Smote methodology to reduce the bias introduced by the class imbalance. This algorithm shifts classification decision boundary to the minority classes that are difficult to learn [33].

### Features selection with Independent Evaluation Criteria for Binary Classification

One of the problems that might occur in the analysis of high-dimensional data is, the curse of dimensionality. It refers to the state that the large number of dimensions of features in the samples is not useful in analyzing and results in a tangible drop in the modeling accuracy. In this situation, not only is increasing the number of dimensions not helpful, it is also destructive. In order to avoid the curse of dimensionality, feature selection was performed. Feature selection is an important step in the classification tasks which by reducing the number of features, enables the classifiers to have a better performance and learn a stronger solution [34,35]. In this study to use the most informative features of QEEG dataset in output classification, our approach for feature selection was based on the ICA. This technique uses the correlation information between each feature and the output based on criterion 1.
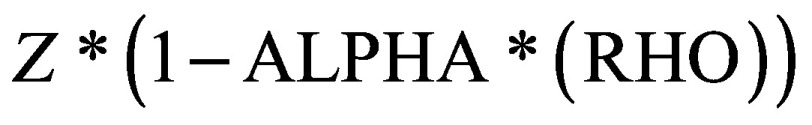



In the above relation, RHO is equal to the average of the absolute values of correlation between candidate feature and previous selected features. The ALPHA represents the controlling element with a default value of 0 (no weight) which sets the weighting factor. The above technique was called and used in MATLAB software as below.
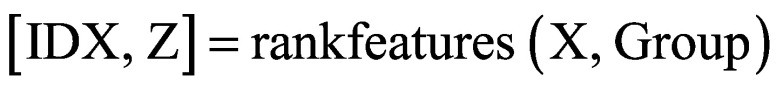



The output of this operation is the IDX and Z vectors. IDX is equivalent to the order of important features and Z represents the value of the correlation coefficient of each attribute to the output.

### Features evaluation with artificial neural network

The extracted outputs in previous phase were the effective features in the classification of IVA-CPT parameters. The extracted features based on the analysis of the ICA had a high correlation with the output of the classification. In this step, the evaluation of selected features was done by a separate classification technique separately. The artificial neural network (ANN) as a simulated model of the human brain model, can detect nonlinear relationships at the desirable level, so it can be an appropriate option for evaluating selected parameters. Hence, 28 neural network models (for each output, a model) based on multilayered perceptron architecture and back-propagation algorithm were designed in order to evaluate the effective QEEG parameters in output classification. Multilayer perceptron as a well-known ANN model is formed of an input, an output and one or more hidden layers. In multilayered perceptron process for determination of weights and biases, input–output data are used [36–39]. Also, the back-propagation technique generates the input forward in a network and in an iterative manner computes the error backward [36,40].

The input of each data model was related to the effective features of the QEEG. Also, each model has one or two hidden layers fit to the input value and the network output is equal to the binarized IVA-CPT parameters. These models fall into the process of learning with the Levenberg–Marquardt algorithm. Data samples were randomly assigned into three sections: train (70%), test (15%) and validation (15%), and participated in the training process. The accuracy of the network on the classification of samples in the test section was considered as a criterion for the optimization of the selected characteristics.

## Results

**Figure 2.**
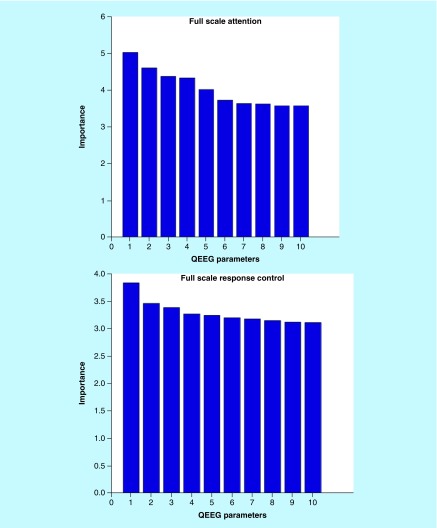
**The importance of top ten QEEG features in the correlation with full scale attention and full scale response control.** QEEG: Quantitative electroencephalography.

The first step in our study was to perform feature selection and ranking. The features of the QEEG dataset were optimized using the ICA. Then obtained optimal subset of QEEG factors was used to gain a better classification result for each 28 output. The report of the analysis was a list of the most priority QEEG parameters which had been ranked based on their importance in the classification of each output. Because of the high numbers of ranked QEEG factors, the top ten factors were considered. Figure 2 shows the diagrams of ranking of top ten QEEG features in the classification of full scale attention and response control. Also the measures, frequency band and brain sites of these ten effective factors are shown in Table 2.

**Table 2.** **The measures, frequency band and brain sites of the top ten QEEG features in correlation with full scale attention and full scale response control.**

**Full scale attention**			**Full scale response control**		

**Measures**	**Freq**	**Sites**	**Measures**	**Freq**	**Sites**
Z-scored FFT amplitude asymmetry	θ	T4, Pz	Z-scored FFT phase lag	α2	Fp2, T4

Z-scored FFT amplitude asymmetry	θ	Fp1, T3	Z-scored FFT phase lag	β2	P3, Cz

Z-scored FFT amplitude asymmetry	θ	F4, T3	Z-scored FFT phase lag	β	Fp1, T5

Z-scored FFT amplitude asymmetry	β1	T3,T6	Z-scored FFT absolute power	α2	Cz

Z-scored FFT amplitude asymmetry	β1	T3,T5	FFT relative power	α2	T5

Z-scored FFT amplitude asymmetry	β1	F4, F7	Z-scored FFT amplitude asymmetry	α	Fz, T3

Z-scored FFT amplitude asymmetry	θ	Fp2, T3	Z-scored FFT absolute power	δ	Pz

Z-scored FFT amplitude asymmetry	θ	F3, T3	Z-scored FFT phase lag high	β	F3, T3

Z-scored FFT amplitude asymmetry	θ	C4, T3	Z-scored FFT phase lag high	β	T5, T6

Z-scored FFT amplitude asymmetry	β1	F3, F7	Z-scored FFT amplitude asymmetry	α1	F7, T3

FFT: Fast fourier transform ; Freq: Frequency.

**Figure 3.**
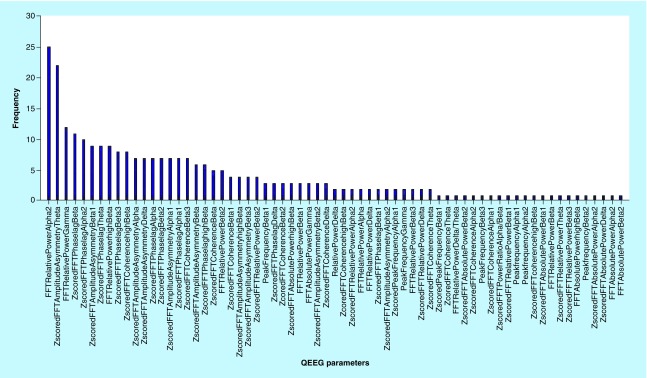
**The frequency of the most important of QEEG features (calculation measures) in the correlation with 28 IVA-CPT parameters.** QEEG: Quantitative electroencephalography.

**Figure 4.**
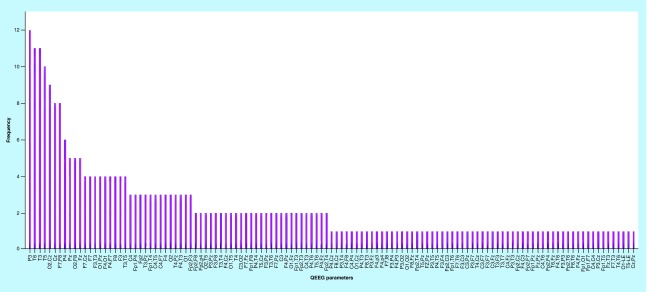
**The frequency of the most important of QEEG features (brain sites) in the correlation with 28 IVA-CPT parameters.** QEEG: Quantitative electroencephalography.

Figures 3 & 4 reflect the frequency of the most important of 280 QEEG features in the correlation with all 28 IVA-CPT parameters. In Figure 3, QEEG features were prioritized according to measures including amplitude asymmetry, phase lag, absolute power, relative power, power ratio, Z score and frequency bands. As is shown in Figure 3, FFT relative power in α2 frequency band has the highest degree of importance while FFT absolute power β2 frequency band was of the least importance. Figure 4 reflects the priority of brain sites in classification of outputs. For considering top ten QEEG factors, P4 was of the highest importance in brain sites. Also, in order to assess the selected features, the ANN was used as the simulation model of brain to classify based on selected features. The results of classification accuracy by using the ANN algorithm for each of outputs are summarized in Table 3. As it is shown from the Figure 1, the classification accuracy of ANN in audio-divided attention, visual speed (attention), auditory speed (attention) and visual stamina (response control) is 99.2, 99.2, 96.8 and 98.9%, respectively. The classification accuracy of other output was 100%.

**Table 3.** **The results of classification accuracy for IVA-CPT parameters using artificial neural network.**

**IVA-CPT parameters**	**ANN accuracy**
Visual-focused attention	100%

Audio-focused attention	100%

Visual-alternating attention	100%

Audio-alternating attention	100%

Visual-sustained attention	100%

Audio-sustained attention	100%

Visual-divided attention	99.2%

Audio-divided attention	99.2%

Visual-selective attention	100%

Audio-selective attention	100%

Visual vigilance (attention)	100%

Visual focus (attention)	100%

Visual speed (attention)	93.7%

Auditory vigilance (attention)	100%

Auditory focus (attention)	100%

Auditory speed (attention)	100%

Full scale attention	100%

Full scale visual attention	100%

Full scale auditory attention	100%

Visual prudence (response control)	98.9%

Visual consistency (response control)	100%

Visual stamina (response control)	99.2%

Auditory prudence (response control)	98.9%

Auditory consistency (response control)	100%

Auditory stamina (response control)	100%

Full scale response control	99.2%

Full scale visual response control	100%

Full scale auditory response control	100%

ANN: Artificial neural network; IVA-CPT: Visual and auditory-continuous performance test

## Discussion

Despite the lack of concrete and fast treatment for ADHD, fortunately many ADHD symptoms including inattention, hyperactivity, impulsivity and response inhibition can be controlled and improved. By identification of deficits in each of these symptoms, therapists can focus on a special cognitive treatment and improve the rehabilitation of children with ADHD. Recently, QEEG clinically has been used in assessment, diagnosis, evaluation and treatment of psychiatric disorders such as ADHD. The QEEG not only has a strong role in differential diagnosis of ADHD, but also has significant heterogeneity among ADHD-diagnosed children and adolescents [41,42].

The present study was performed to identify the most effective features of QEEG in the determination of IVA-CPT parameters (attention and response control) in children with ADHD. The study was conducted based on using the Independent Evaluation Criterion for Binary Classification to analyze the QEEG parameters. Also, by applying ANN as an optimum technique to evaluate the QEEG features in output classification, the results of features selection were confirmed.

Previous researches showed the children with ADHD compared with normal subjects, due to the reduced cortical activity, have EEG differences in brain electrical activity, particularly the increase in δ-band frequency in the fronto-central region. They also pointed the high accuracy and sensitivity of QEEG in differentiation of ADHD from the normal group [5,6,25,41–56]. Some other studies highlighted the changes of QEEG patterns in behavioral symptoms and deficit in cognitive skills such as attention, reaction time and impulsivity [5,6,25,46].

Related to such research, the present study focused on ADHD and highlighted that the EEG patterns are also different among ADHD samples. These variations are due to the wide heterogeneity and heterogeneity of ADHD symptoms and also the severity of them [57]. Our analysis, by focusing on attention and response control as two main ADHD symptoms, confirmed that the ADHD subjects, based on receiving scores in attention and response control by using IVA-CPT, showed different patterns of electroencephalography. Such variations were shown even in two visual and auditory dimensions of each IVA-CPT parameter. For instance, in visual-divided attention, the most effective QEEG feature belonged to FFT relative power in the α-frequency band in T4 site while in audio-divided attention; Z-scored FFT coherence in β-frequency band in O2 and Cz sites has the high priority.

Epileptic patterns, increase in the power of the δ- and θ-frequency bands, elevated level of δ/β ratio and reduction in β- and α-frequency bands particularly in posterior regions, are the most consistent findings that have been observed in many studies related to the analysis of EEG in children with ADHD [4,7,14,41,58–62]. In support of previous research, this study highlighted and ranked the brain wave frequency bands and also brain sites to indicate the impact of each of these brain wave frequency bands and sites on the IVA-CPT parameters.

According to Hillard *et al*. [63], change in EEG bands’ relative power will be accompanied with inattention. The study of Dongen-Boomsma *et al*. uncovered the correlation of δ/α and δ/β ratios and the response time [64]. The study of Ogrim *et al*. supports the results of the study of Dongen-Boomsma *et al*. and also highlighted that δ-β ratio may demonstrate the increase in impulsivity [56]. Consistent with these studies, our finding showed the FFT relative power with the highest frequency, as a main measure in correlation with overall IVA-CPT parameters, although, in the analysis of each 28 IVA-CPT parameters separately, such result was not supported. As such, the highest measures in full attention and full response control were Z-scored FFT amplitude asymmetry and Z-scored FFT phase lag, respectively.

Some studies investigated the correlation between EEG and neuropsychological test. Koehler *et al*. indicated a positive relation between δ- frequency band and inattention scores on the self-report scale of individuals with ADHD. Also, the study of Clarke *et al*. showed the relation between EEG features and Conners’ Rating Scale. The result of these studies confirmed the correlation of the δ-frequency band in frontal region with inattention and also the correlation of δ-β ratio with hyperactivity–impulsivity [65,66]. The study of Kim *et al*. was one of the most relevant studies regarding the relationship between IVA-CPT and EEG. In this study, by correlating between the IVA-CPT and EEG parameters, the EEG power spectrum and the δ-phase γ-phase amplitude measurement data were analyzed [22]. Similar to such studies, current research, by designing a model of correlation between QEEG features and IVA-CPT, determined the most priority of QEEG features in the classification of IVA-CPT parameters. According to previous studies, the age and IQ can affect the power of EEG frequency bands [50,67]. The present study analyzed the data of all ADHD subjects were included in the study to correlate between QEEG and IVA-CPT, and no intervention was done on ADHD samples. The impact of age and IQ and other confounding factors on EEG patterns, was not considered. Also, another limitation of this study was the insufficiency of the sample size compared with the number of QEEG features. Thus, the results must be considered with caution.

## Conclusion & future perspective

Today, it is accepted that EEG provides a potential outlook of the neurophysiology of the brain. EEG studies on children with ADHD are performed to explore the different aspects of brain functions in them [21]. In the present study, a model for correlation between QEEG and IVA-CPT was designed. By developing Clinical Decision Support System based on the designed model and using the system in clinical trials and research studies, therapists could be able to determine the existence or absence of defects in IVA-CPT parameters related to attention and response control, relying on QEEG. As well, they can concentrate on specific cognitive skill such as attention to suggest an optimal approach for rehabilitate it. We propose further researches about the correlation between QEEG and neuropsychological tests such as IVA-CPT with more ADHD subjects.

**Summary points**

**Background**

- This study identify the most prominent features of quantitative electroencephalography that affect attention and response control parameters in the children and adolescents with attention deficit hyperactivity disorder.

**Materials & methods**

- The feature selection was done by Independent Evaluation Criterion for Binary Classification and then was evaluated by using the artificial neural network.

**Results**

- The highest rank of QEEG features in each Integrated Visual and Auditory-Continuous Performance Test parameters were uncovered.

**Discussion**

- The designed model could be used in other domains which the scanning and checking of the attention and response control parameters, is important.
